# Editorial: Metagenomic approach for exploration of antimicrobial resistance in uncultivated microbiota

**DOI:** 10.3389/fmicb.2025.1633252

**Published:** 2025-06-17

**Authors:** Ali H. A. Elbehery, Karsten Becker, Abhijeet Mishra, Razi Ahmad

**Affiliations:** ^1^Department of Microbiology and Immunology, Faculty of Pharmacy, University of Sadat City, Sadat City, Egypt; ^2^Friedrich Loeffler-Institute of Medical Microbiology, University Medicine Greifswald, Greifswald, Germany; ^3^Shivaji College, University of Delhi, New Delhi, India; ^4^Indian Institute of Technology Delhi, New Delhi, India

**Keywords:** metagenomics, antimicrobial resistance (AMR), bioinformatics and computational biology, diagnostics—clinical characteristics, resistance genes

Antimicrobial resistance (AMR) has emerged as one of the most pressing and intricate health concerns of the twenty-first century. Given the projected 1.27 million annual fatalities directly linked to AMR (Murray et al., 2022; Naghavi et al., 2024) and millions more impacted worldwide, the necessity for a comprehensive understanding of resistance dynamics through innovative tools is paramount. Conventional AMR research has predominantly depended on culture-based methodologies targeting therapeutically relevant bacteria. While this methodology continues to be important for routine therapeutic purposes in human and veterinary medicine, especially for detecting unknown and phenotypic resistance patterns, it has become clearer that the majority of microbial life cannot be cultivated under typical laboratory settings, also known as the “great plate count anomaly” (Staley and Konopka, 1985). This disparity has prompted a transition toward metagenomics as an effective approach to identify resistance elements in previously unattainable microbiota.

Metagenomic methodologies enable direct acquisition and analysis of genetic data from diverse samples without prior culture requirements (Handelsman, 2004). This enables researchers to thoroughly examine the prevalence, diversity, and mobility of antibiotic resistance genes (ARGs) and their correlation with mobile genomic elements (MGEs). This Research Topic brings together eight original contributions that collectively highlight the breadth and depth of current metagenomic approaches in AMR research. These studies span diverse ecosystems, from hospital settings and host-associated microbiota to rivers, ponds, and agricultural soils, offering valuable insights into the evolution, spread, and ecological context of AMR, with a significant focus on One Health integration.

A significant work in this Research Topic by Tian and Zhang, analyzed 1,382 intestinal metagenomes from Chinese individuals and discovered more than 638,000 non-redundant MGEs. The research indicated a significant correlation between socioeconomic parameters, such as the country's economic output per person (GDP per capita), and the abundance of MGE and ARG, implying that industrialization may unintentionally promote the dissemination of antimicrobial resistance via modified gut microbiomes. This demonstrates how metagenomic surveillance might uncover intricate relationships between human development and the dynamics of bacterial resistance.

In addition, Chekole et al., employed a One Health paradigm in Ethiopia to examine *E. coli* isolates from calves, humans, and the environment. Utilizing whole genome sequencing and metagenomic annotation, they revealed pervasive antimicrobial resistance determinants and mobile genetic elements, including Tn3, Int1, and dhfr7, with 95% of antibiotic resistance genes detected across various sources. This underscores how cross-reservoir transmission, facilitated by horizontal gene transfer, influences the resistome in resource-limited, high-burden environments.

Plasmid-mediated gene transfer is a primary catalyst for the spread of resistance. Liu et al., documented the initial detection of an IncHI5-like plasmid concurrently containing *bla*_NDM − 1_ and *bla*_OXA − 1_ in *Klebsiella pneumoniae* from clinical specimens in China. The plasmid displayed a hybrid configuration containing many resistance genes, prompting considerable clinical apprehension on the emergence of pan-resistant diseases. The high-resolution plasmid study by metagenomics and comparative genomics highlights the evolutionary capacity of mobile genetic elements in hospital-acquired infections.

Methodological advances are also showcased in ARGContextProfiler. Moumi et al., presented an innovative pipeline that extracts and evaluates ARGs within their genomic settings utilizing assembly graphs. This technique distinguishes ARGs integrated into chromosomes from those linked to MGEs, yielding more precise estimates of resistance mobility. Through rigorous validation on both actual and synthetic datasets, ARGContextProfiler improves surveillance capabilities and facilitates precise tracking of clinically significant ARGs.

Environmental resistomes constitute an understudied, yet crucial aspect of antimicrobial resistance evolution. Wang et al., performed an extensive metagenomic examination of the Holtemme river in Germany, illustrating the significant impact of anthropogenic activities, such as wastewater discharge, on the quantity and diversity of ARGs. Their research identified ARGs, such as OXA-4, in plasmids of environmental bacteria like *Thiolinea (Thiothrix) eikelboomii*, highlighting the role of environmental microbiota as reservoirs and vectors for ARG transmission.

Moreover, Huang et al., investigated the effects of ozone nanobubble treatments in aquaculture. Employing shotgun metagenomics, they noted an increased prevalence of antibiotic resistance genes, especially those associated with efflux-mediated resistance, following treatment. This study warns that disinfection methods, while intended to decrease infections, may unintentionally promote resistant bacteria, emphasizing the need for a careful assessment of the use of disinfectants in aquatic environments and its influence on AMR.

You et al., investigated agricultural methods, evaluating the impact of different nitrogen fertilizers on soil antibiotic resistance gene profiles. Notably, although soil bacterial communities varied with fertilizer type, key ARGs exhibited relative stability, suggesting a minimal effect on the resistome. Nevertheless, they discovered a correlation between nitrogen-cycling genes and ARGs, indicating potential indirect selection forces that merit further investigation.

The post-COVID-19 period has significantly transformed resistance settings. Debnath et al., reported a 22% frequency of azithromycin resistance among clinical isolates in India, primarily attributed to plasmid-mediated *mphA* genes. This corresponds with global trends of increasing macrolide resistance following the pandemic and highlights the pressing necessity for coordinated antimicrobial resistance surveillance programs.

These contributions collectively exemplify the forefront of metagenomics-driven antimicrobial resistance research. They present persuasive evidence that AMR is not limited to pathogenic, culturable bacteria but is ingrained in the extensive, predominantly uncultivated microbial realm across several ecosystems. Metagenomics allows researchers to identify new resistance determinants, monitor their dissemination, and comprehend their contextual relationships with mobile genetic elements, infections, and environmental reservoirs.

Nonetheless, obstacles persist. The standardization of sampling techniques, bioinformatics pipelines, and reference databases is crucial for ensuring comparability between studies. The incorporation of metagenomics into clinical diagnostics necessitates validation mechanisms and quality management measures to convert research discoveries into implementable public health measures.

In conclusion, the studies assembled under this Research Topic showcase the transformative potential of metagenomics in deciphering the complex ecology and evolution of the wide diversity of microbial strategies for the development of antibiotic and other resistance mechanisms and, in the case of MGEs, their transmission within and between microbial species. By bridging clinical, environmental, and technological domains, they advance our understanding and offer practical tools to confront the AMR crisis. The metagenomic lens not only reveals the hidden resistome, but also empowers global efforts to curb its expansion in a One Health framework.
